# Patterns and trends of hepatitis C virus infection in Jordan: an observational study

**DOI:** 10.3389/fpubh.2023.1280427

**Published:** 2023-12-07

**Authors:** Issa Abu-Dayyeh, Hiam Chemaitelly, Mohammad Ghunaim, Thaer Hasan, Amid Abdelnour, Laith J. Abu-Raddad

**Affiliations:** ^1^Department of Research and Development, Biolab Diagnostic Laboratories, Amman, Jordan; ^2^Infectious Disease Epidemiology Group, Weill Cornell Medicine-Qatar, Cornell University, Doha, Qatar; ^3^World Health Organization Collaborating Centre for Disease Epidemiology Analytics on HIV/AIDS, Sexually Transmitted Infections, and Viral Hepatitis, Weill Cornell Medicine–Qatar, Cornell University, Qatar Foundation – Education City, Doha, Qatar; ^4^Department of Population Health Sciences, Weill Cornell Medicine, Cornell University, New York, NY, United States; ^5^Department of Public Health, College of Health Sciences, QU Health, Qatar University, Doha, Qatar; ^6^College of Health and Life Sciences, Hamad bin Khalifa University, Doha, Qatar

**Keywords:** hepatitis C virus, prevalence, incidence, viremic rate, cohort study, Jordan, Middle East and North Africa

## Abstract

**Background:**

Hepatitis C virus (HCV) infection levels in Jordan remain uncertain. No HCV national population-based survey has ever been conducted in the country. To meet the World Health Organization’s target of reducing HCV incidence to ≤5 per 100,000 people per year by 2030, it is essential to determine the infection levels, identify affected individuals and populations, and provide appropriate treatment using direct-acting antivirals to individuals carrying the virus.

**Methods:**

The study utilized the HCV testing database of 28,798 attendees of Biolab Diagnostic Laboratories in Jordan, covering the period from January 19, 2010, to May 26, 2023. Cross-sectional and cohort study analyses were conducted, including estimating HCV antibody (Ab) prevalence, examining associations with HCV Ab positivity, determining the HCV viremic rate, and estimating HCV incidence rate using a retrospective cohort study design.

**Results:**

A total of 27,591 individuals, with a median age of 31.3 and 52.9% being females, underwent HCV Ab testing, while 1,450 individuals, with a median age of 42.2 and 32.8% being females, underwent HCV RNA PCR testing. The study sample HCV Ab prevalence was 4.0% (95% CI: 3.7–4.2%). After applying probability weights, the weighted HCV Ab prevalence was 5.8% (95% CI: 4.6–7.3%). Age was strongly associated with HCV Ab positivity, particularly among individuals aged 50 years or older, who had 10-fold higher odds of being HCV Ab positive compared to those aged 10–19 years. Males had 2.41-fold higher odds of testing positive for HCV Ab compared to females. The HCV viremic rate was 54.1% (95% CI: 43.0–65.0%). The cumulative incidence of HCV infection, after 5 years of follow-up, was estimated to be 0.41% (95% CI: 0.17–0.99%). The HCV incidence rate was calculated at 1.19 per 1,000 person-years (95% CI, 0.50–2.87).

**Conclusion:**

Prevalence and incidence of HCV infection were substantial, estimated at ~5% and 1 per 1,000 person-years, respectively, and highlighting the presence of core groups actively engaged in the virus’ acquisition and transmission. The high observed viremic rate indicates the need for expanding HCV treatment efforts to effectively control HCV transmission in Jordan. Utilizing quality diagnostic laboratories and innovative testing strategies is key to identifying infection carriers and facilitating linkage to treatment and care.

## Introduction

According to the Global Burden of Disease Study, viral hepatitis is the 7th major cause of death globally (1). Hepatitis C virus (HCV) infection is responsible for approximately half of these deaths (1). HCV is a blood-borne virus, and its transmission can be substantially reduced through proper preventive measures (2). Contracting HCV can lead to various health complications, including acute hepatitis, fibrosis, cirrhosis, and potentially liver cancer (3, 4).

The development of highly effective direct-acting antivirals (DAAs) has been a breakthrough in the treatment and control of HCV infection (5–7). DAAs have been associated with significant reductions in the burden and cost of managing liver-related conditions, suggesting a potential for eliminating HCV infection as a public health threat (5–7).

The availability and recent affordability of DAAs, even in resource-limited countries where the cost can be below $100 for a complete treatment course (8, 9), have prompted the World Health Organization (WHO) to advocate for ambitious global targets for the diagnosis, treatment, and cure of viral hepatitis. This initiative represents a significant momentum towards the goal of eliminating HCV infection by 2030 (10, 11).

Of all regions, the Middle East and North Africa (MENA) region is most affected by HCV infection (1, 12), although the prevalence of infection varies across countries. Jordan, a MENA country with a population of 11 million, is believed to have a low prevalence of HCV (13). However, unlike MENA countries including Egypt (14–16), Pakistan (17, 18), and Libya (19), there has never been a nationally representative population-based survey conducted to assess the level of infection in Jordan. Based on available data on HCV antibody (Ab) prevalence, a meta-analysis estimated the HCV Ab prevalence in Jordan at only 0.3% (13), which is one of the lowest rates worldwide (20, 21).

This study aimed to address the lack of current and detailed knowledge on HCV infection in Jordan by analyzing a large HCV testing database that covers all major urban centers across the country. Through descriptive and analytical study designs, the database was investigated to gain insights into the epidemiology of HCV infection, identify patterns and associations related to the infection, and provide evidence for shaping public health policy, prioritizing programming initiatives, and allocating resources effectively within the country.

## Methods

### Study population and data sources

This study was conducted in Jordan using the HCV testing database of Biolab Diagnostic Laboratories (Biolab) from January 19, 2010, to May 26, 2023. Biolab is a group of nationally and internationally accredited private medical diagnostic laboratories established in 2001, offering a comprehensive range of high-volume medical laboratory services within Jordan (22). Biolab operates 27 branches across Jordan, covering all major urban centers in the country. This extensive network allows Biolab to efficiently serve a large number of walk-in patients, offering testing services to a wide range of individuals. Biolab also receives samples referred by other diagnostic laboratories in Jordan or neighboring countries, further contributing to its wide testing coverage.

The database investigated in this study encompasses all HCV Ab testing and HCV polymerase chain reaction (PCR) testing conducted at Biolab, across all of its branches, since the implementation of its digital Laboratory Information Management System (LIMS). The extracted data includes information such as sex, age, location of the test (specific Biolab branch), date of the test, type of test (Ab or PCR), HCV antibody titer (optical density value) result from the Ab testing, and the HCV ribonucleic acid (RNA) viral load result from the PCR testing. The database does not contain any additional characteristics or identifiers beyond these mentioned variables.

The specific reasons for testing are variable and were not captured in the data. However, based on anecdotal evidence and the context surrounding testing practices (23), the study population can be described as individuals who demonstrate a tendency to seek HCV testing or as attendees of a diagnostic laboratory for HCV testing.

The study population may include individuals who were referred for testing by their healthcare providers (23), individuals showing clinical symptoms or signs consistent with a hepatitis infection, or individuals with known risk factors for HCV exposure such as patients undergoing hemodialysis (24), individuals who engage in drug injection (25, 26), individuals who have received blood transfusions (27), or individuals who have experienced needle-stick injuries (27). The tested population may also include individuals undergoing testing because of a routine testing requirement, such as for employment, travel, or education abroad, or as part of a set panel of tests recommended for specific screening, such as for sexually transmitted infections. The testing population may further include individuals who were suspected to be infected due to the diagnosis of HCV infection in a close relative or individuals who proactively chose to get tested to determine their infection status, particularly considering the recent availability of effective HCV treatments.

### Study designs and analyses

Following the exclusion of samples coming from outside of Jordan, a series of analyses was performed using complete information for outcomes of interest. Descriptive characteristics of the testing samples were provided, and HCV Ab prevalence was calculated for the overall sample, as well as using 2-year time intervals. Only the first HCV Ab test for each individual in each considered time interval was included in this analysis.

Furthermore, HCV Ab prevalence was determined by applying probability weights based on factors including sex, age, and location (specifically, the Jordanian governorate, which represents the main administrative unit for regions in Jordan). To explore associations with HCV Ab positivity, both univariable and multivariable regression analyses were conducted. These regression analyses incorporated all individuals with at least one HCV Ab test.

The HCV viremic rate, which indicates the proportion of individuals previously infected with HCV who continue to be chronically infected without clearing the infection naturally or through treatment, was calculated. The viremic rate was estimated by determining the proportion of individuals who tested positive for HCV RNA by PCR among those who tested positive for HCV antibodies (28, 29). For this analysis, only individuals who had both a positive HCV Ab test and an HCV RNA PCR test, regardless of the PCR test outcome, were included. Only the first PCR test result for each individual was included in this analysis.

The HCV incidence rate was estimated among the study population using a retrospective cohort study design. The cohort study consisted of individuals who underwent at least two HCV Ab tests, with the first test result being negative for HCV Ab. Individuals in the study cohort were followed from the date of the first HCV Ab negative test up until an HCV Ab positive test. Otherwise, they were censored at the date of their last recorded HCV Ab test. Thus, individuals were followed up until the occurrence of either an HCV Ab positive test or their last recorded HCV Ab test. The incident infection event was assumed to have taken place at the midpoint between the dates of the positive Ab test and the preceding negative Ab test.

### Laboratory methods

Testing for HCV antibodies was conducted using a fully automated electrochemiluminescence immunoassay (Elecsys Anti-HCV immunoassay; Roche Diagnostics, Penzberg, Germany) on a Cobas 6,000 or Cobas Pro platform following the manufacturer’s recommendations. Serum samples with a signal/cut-off (optical density) ratio (s/co; i.e., antibody titers) <0.9 were categorized as non-reactive, those between 0.9 and 1 were considered indeterminate, and samples with a ratio of ≥1.0 were classified as reactive.

All indeterminate and reactive specimens were confirmed using the LIAISON XL chemiluminescence system for the detection of anti-HCV antibodies (LIAISON XL Murex HCV Ab, DiaSorin SpA, Saluggia, Italy) as part of a set algorithm (Supplementary Figure S1). In cases of a discrepancy, a second sample was requested from the patient for retesting. On rare occasions when the discrepancy persisted and uncertainty arose regarding the test outcome, the sample underwent further examination. It was then sent to either the National Reference Laboratory of the Ministry of Health or to an internationally accredited diagnostic laboratory, whether located within or outside Jordan, for final testing and confirmation of the outcome.

HCV RNA detection was performed by an automated real-time reverse transcription PCR (rRT-PCR) using quantitative HCV RNA assays including COBAS Taqman (Roche Diagnostics, United States; lower-limit quantitation of 25 IU/mL) from 2010 to May 2021, and GeneXpert (Cepheid, USA; lower-limit quantitation of 10 IU/mL) thereafter. Both assays were used according to the manufacturers’ instructions. Laboratory testing was conducted at Biolab Diagnostic Laboratories following standardized protocols.

### Oversight

The institutional review boards at Biolab and Weill Cornell Medicine–Qatar approved this retrospective study with a waiver of informed consent. The cross-sectional and cohort components of the study were reported according to the Strengthening the Reporting of Observational Studies in Epidemiology (STROBE) guidelines (Supplementary Tables S1, S2, respectively).

### Statistical analysis

The characteristics of the study population and the incidence study cohort were described using frequency distributions and measures of central tendency. The prevalence of HCV Ab in the study sample was determined as the proportion of individuals who tested positive for HCV Ab among those who underwent HCV Ab testing.

Weighted HCV Ab prevalence for the study population was calculated by applying probability weights to adjust for the unequal selection of participants with respect to the sex, age, and governorate distribution of the total population of Jordan. This adjustment was necessary to ensure that the estimated HCV Ab prevalence better represented the characteristics of the study population from which the testing sample was derived. Probability weights were calculated using the distribution of the population of Jordan by sex and age in 2021 per the United Nations World Population Prospects database (30) and the distribution of the population of Jordan by governorate as provided by Jordan’s 2015 General Population and Housing Census (31).

Associations with HCV Ab positivity were examined through Chi-square tests and univariable logistic regression analyses. Covariates with a value of p of ≤0.2 in the univariable regression analysis were included in the multivariable model. Covariates with a value of p of ≤0.05 in the multivariable analysis were considered to have statistically significant evidence for an association with the outcome. Results were reported in terms of odds ratios (ORs), adjusted ORs (AORs), 95% confidence intervals (CIs), and corresponding value of ps.

The cumulative incidence of HCV infection among the incidence study cohort, defined as the proportion of individuals at risk whose primary endpoint during the follow-up period was an HCV Ab positive test, was estimated using the Kaplan–Meier estimator method (32). The incidence rate of infection among the incidence study cohort, defined as the number of identified infections divided by the number of person-years contributed by all individuals in the cohort, and the corresponding 95% CI, were estimated using a Poisson log-likelihood regression model with the Stata 17.0 *stptime* command. Interactions were not considered. Statistical analyses were conducted using Stata/SE version 17.0 (Stata Corporation, College Station, TX, United States).

## Results

### Study samples

**Figure 1:**
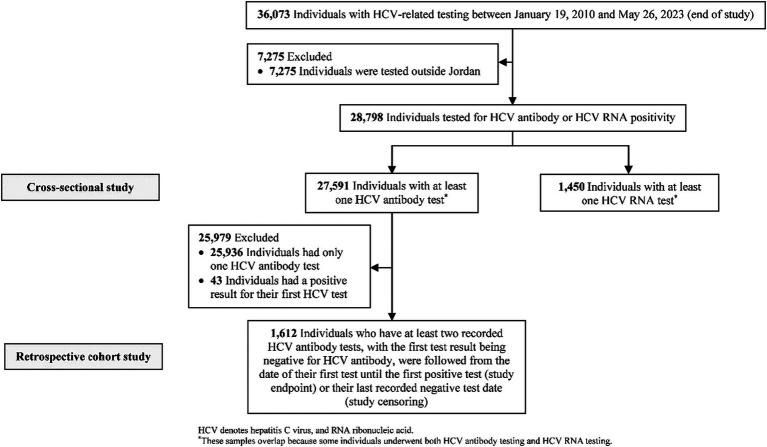
Flowchart describing the population selection process.

Figure 1 illustrates the process of selecting the study population. A total of 28,798 individuals were included in various analyses conducted in the study. Table 1 provides a description of the characteristics of the 27,591 individuals who underwent at least one HCV Ab test. The median age was 31.3 years, with 52.9% of the sample being females. The vast majority of individuals tested were from the Amman governorate. On average, 2,133 HCV Ab tests were conducted annually, and the frequency of tests remained relatively consistent over the years. There was some variation in the number of HCV Ab tests per person, with only 6% of the sample undergoing more than one test during the study duration.

**Table 1:** Characteristics of individuals with at least one HCV antibody test.

Characteristics	N (%) N = 27,591
Median age (IQR)—years	31.3 (25.3–40.4)
Age (years)	
0–9	321 (1.2)
10–19	1,051 (3.8)
20–29	10,465 (37.9)
30–39	8,600 (31.2)
40–49	3,537 (12.8)
50–59	1,916 (6.9)
60–69	1,012 (3.7)
70+	689 (2.5)
Sex	
Female	14,589 (52.9)
Male	13,002 (47.1)
Governorate	
Amman	25,784 (93.5)
Aqaba	95 (0.3)
Balqaa	387 (1.4)
Irbid	437 (1.6)
Karak	8 (0.03)
Maan	148 (0.5)
Madaba	25 (0.09)
Tafila	84 (0.3)
Zarqa	623 (2.3)
Year of first HCV Ab test	
2010	667 (2.4)
2011	841 (3.0)
2012	1,254 (4.5)
2013	1,756 (6.4)
2014	2,203 (8.0)
2015	2,503 (9.1)
2016	2,544 (9.2)
2017	2,614 (9.5)
2018	2,273 (8.2)
2019	2,490 (9.0)
2020	2,232 (8.1)
2021	2,358 (8.6)
2022	2,485 (9.0)
2023	1,371 (5.0)
Proportion with	
Only 1 test	25,936 (94.0)
2 tests	1,311 (4.8)
3 tests	225 (0.8)
4 tests	62 (0.2)
5+ tests	57 (0.2)
HCV status	
At least one positive test	1,101 (4.0)
All negative tests	26,490 (96.0)

HCV denotes hepatitis C virus and IQR, interquartile range.

Supplementary Table S3 describes the characteristics of individuals who had at least one HCV RNA PCR test. The median age was 42.2 years, 32.8% of the sample were females, and the vast majority of those tested were from the Amman governorate. On average, 120 HCV PCR tests were performed annually with minimal variability in frequency over the years. There was some variation in the number of HCV PCR tests per person, with 7% of the sample having more than one test over the study duration.

### HCV ab prevalence

**Figure 2:**
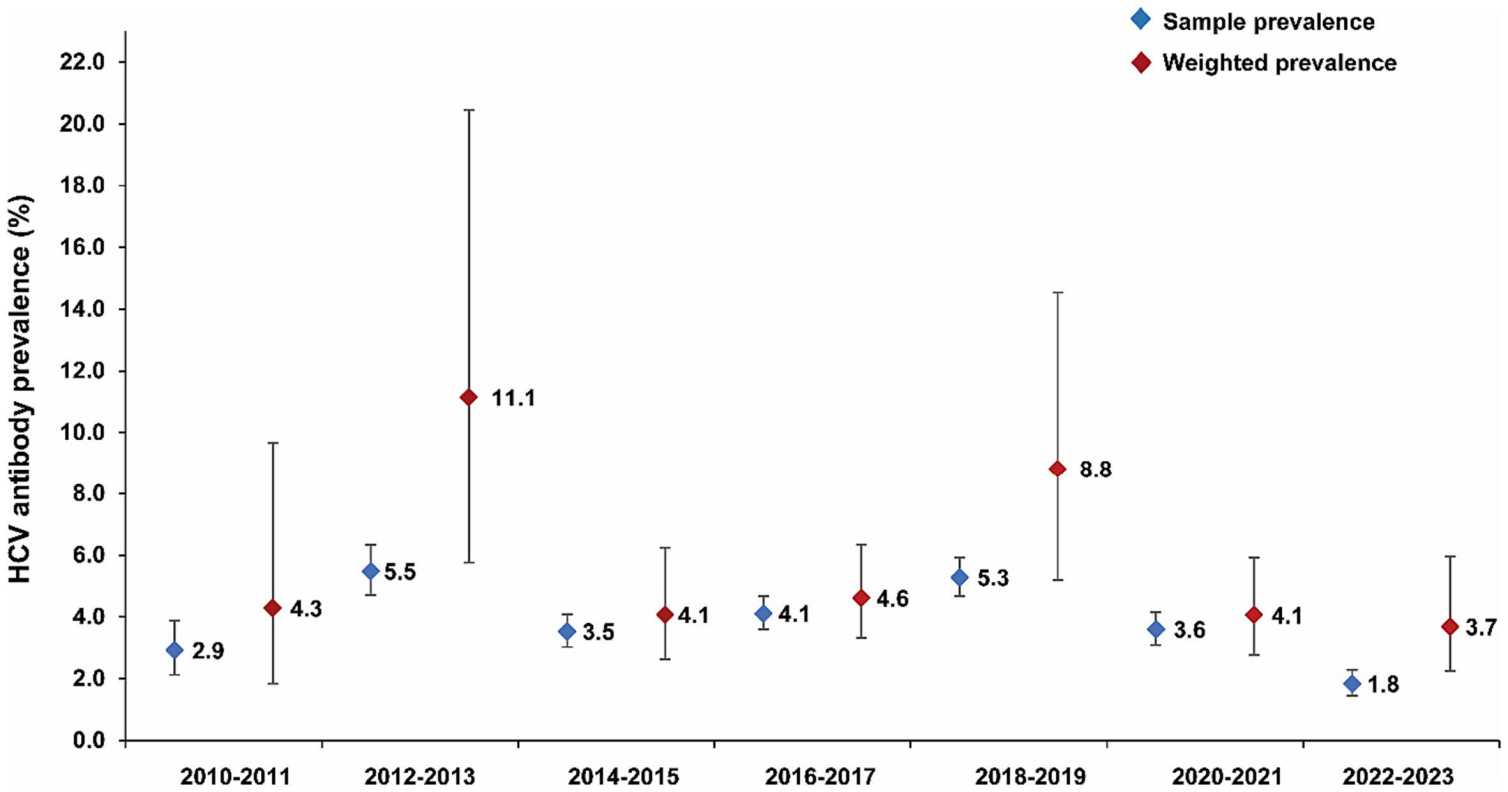
HCV antibody prevalence over the study duration.

Out of the 27,591 individuals who underwent an HCV Ab test, 1,101 individuals tested positive, resulting in a sample prevalence of 4.0% (95% CI: 3.7–4.2%). After applying the probability weights to account for variations in the sex, age, and governorate distribution, the weighted HCV Ab prevalence was estimated at 5.8% (95% CI: 4.6–7.3%). Figure 2 shows HCV Ab prevalence by 2-year time intervals. Supplementary Figure S2 displays the distribution of antibody titers (optical density values) among the individuals who tested positive for HCV Ab, with a median of 50.7 (interquartile range, IQR: 32.3–87.1).

### Associations with HCV ab positivity

Table 2 provides an overview of the crude and adjusted ORs describing the associations with being HCV Ab positive. Age was found to have a strong association with HCV Ab positivity, with the AOR increasing with older age. The association was particularly pronounced among individuals aged 50 years or older, who had 10-fold higher odds of being HCV Ab positive compared to those in the age group of 10–19 years.

**Table 2:** Key associations with being HCV antibody positive.

Characteristics	Tested	HCV antibody-positive		Univariable regression analysis				Multivariable regression analysis^a^	
	N	N (%)	*p*-value	OR (95% CI)	*p*-value	F test *p*-value^b^	R^2^ (%)	AOR (95% CI)	*p*-value^c^
Age (years)			<0.001			<0.001	6.2		
10–19^d^	1,051	12 (1.1)		1.00				1.00	
0–9	321	6 (1.9)		1.65 (0.61–4.43)	0.321			1.47 (0.54–3.95)	0.448
20–29	10,465	153 (1.5)		1.28 (0.71–2.32)	0.406			1.55 (0.85–2.80)	0.150
30–39	8,600	337 (3.9)		3.53 (1.98–6.30)	<0.001			4.05 (2.26–7.25)	<0.001
40–49	3,537	221 (6.2)		5.77 (3.21–10.36)	<0.001			5.74 (3.19–10.33)	<0.001
50–59	1,916	197 (10.3)		9.92 (5.51–17.86)	<0.001			10.24 (5.67–18.49)	<0.001
60–69	1,012	119 (11.8)		11.54 (6.33–21.03)	<0.001			12.32 (6.73–22.52)	<0.001
70+	689	56 (8.1)		7.66 (4.07–14.40)	<0.001			8.22 (4.36–15.51)	<0.001
Sex			<0.001			<0.001	2.9		
Female	14,589	321 (2.2)		1.00				1.00	
Male	13,002	780 (6.0)		2.84 (2.49–3.24)	<0.001			2.41 (2.11–2.76)	<0.001
Governorate			<0.001			<0.001	0.8		
Amman	25,784	963 (3.7)		1.00				1.00	
Aqaba	95	7 (7.4)		2.05 (0.95–4.44)	0.068			2.34 (1.05–5.20)	0.037
Balqaa	387	18 (4.7)		1.26 (0.78–2.03)	0.347			1.45 (0.89–2.37)	0.135
Irbid	437	38 (8.7)		2.45 (1.75–3.45)	<0.001			2.55 (1.79–3.62)	<0.001
Karak	8	1 (12.5)		3.68 (0.45–29.96)	0.223			6.62 (0.75–58.26)	0.088
Maan	148	11 (7.4)		2.07 (1.12–3.84)	0.021			1.65 (0.87–3.12)	0.124
Madaba	25	6 (24.0)		8.14 (3.24–20.43)	<0.001			7.31 (2.77–19.27)	<0.001
Tafila	84	11 (13.1)		3.88 (2.05–7.34)	<0.001			3.66 (1.88–7.11)	<0.001
Zarqa	623	46 (7.4)		2.05 (1.51–2.79)	<0.001			2.15 (1.56–2.95)	<0.001
Proportion with			0.209			0.164	0.1		
Only 1 test	25,936	1,053 (4.1)		1.00				1.00	
2 tests	1,311	36 (2.7)		0.67 (0.48–0.93)	0.019			0.69 (0.49–0.97)	0.033
3 tests	225	8 (3.6)		0.87 (0.43–1.77)	0.703			0.83 (0.40–1.70)	0.607
4 tests	62	2 (3.2)		0.79 (0.19–3.23)	0.740			0.62 (0.15–2.58)	0.509
5+ tests	57	2 (3.5)		0.86 (0.21–3.53)	0.833			0.59 (0.14–2.45)	0.466
Year of the HCV test^e^	27,591	1,101 (4.0)	NA	0.97 (0.96–0.99)	0.002	0.002	0.1	0.98 (0.96–0.99)	0.011

AOR denotes adjusted odds ratio, CI, confidence interval, NA, not applicable, and OR, odds ratio.

a The multivariable model explained 9.1% of the total variability in HCV prevalence.

b Covariates with *p*-value ≤ 0.2 in the univariable analysis were included in the multivariable analysis.

c Covariates with *p*-value ≤ 0.05 in the multivariable analysis were considered as showing strong evidence for an association with HCV antibody positivity.

d The 10–19 years age group was chosen as a reference because of the small sample size of those in the 0–9 years age group.

e The year of the HCV test was the year of the first positive test for HCV antibody-positive individuals and the year of the first negative test for HCV antibody-negative individuals.

Males had 2.41-fold (95% CI: 2.11–2.76) higher odds of being HCV Ab positive compared to females. HCV Ab tests conducted outside the Amman governorate were associated with higher odds of testing positive. Individuals who had more than one HCV Ab test were less likely to test positive for HCV Ab. There was an indication for a decline in HCV Ab test positivity over time by an AOR of 0.98 per year (95% CI: 0.96–0.99).

### HCV viremic rate

Supplementary Figure S3 displays the distribution of HCV RNA viral load among the 645 individuals who had at least one positive HCV PCR test result. The median viral load was 175,500 IU/ml (IQR: 9,910-1,225,000). Out of 85 individuals who had records for both an HCV Ab positive test and a PCR test, 46 tested positive for HCV RNA, resulting in an HCV viremic rate of 54.1% (95% CI: 43.0–65.0%).

### HCV incidence rate

**Figure 3:**
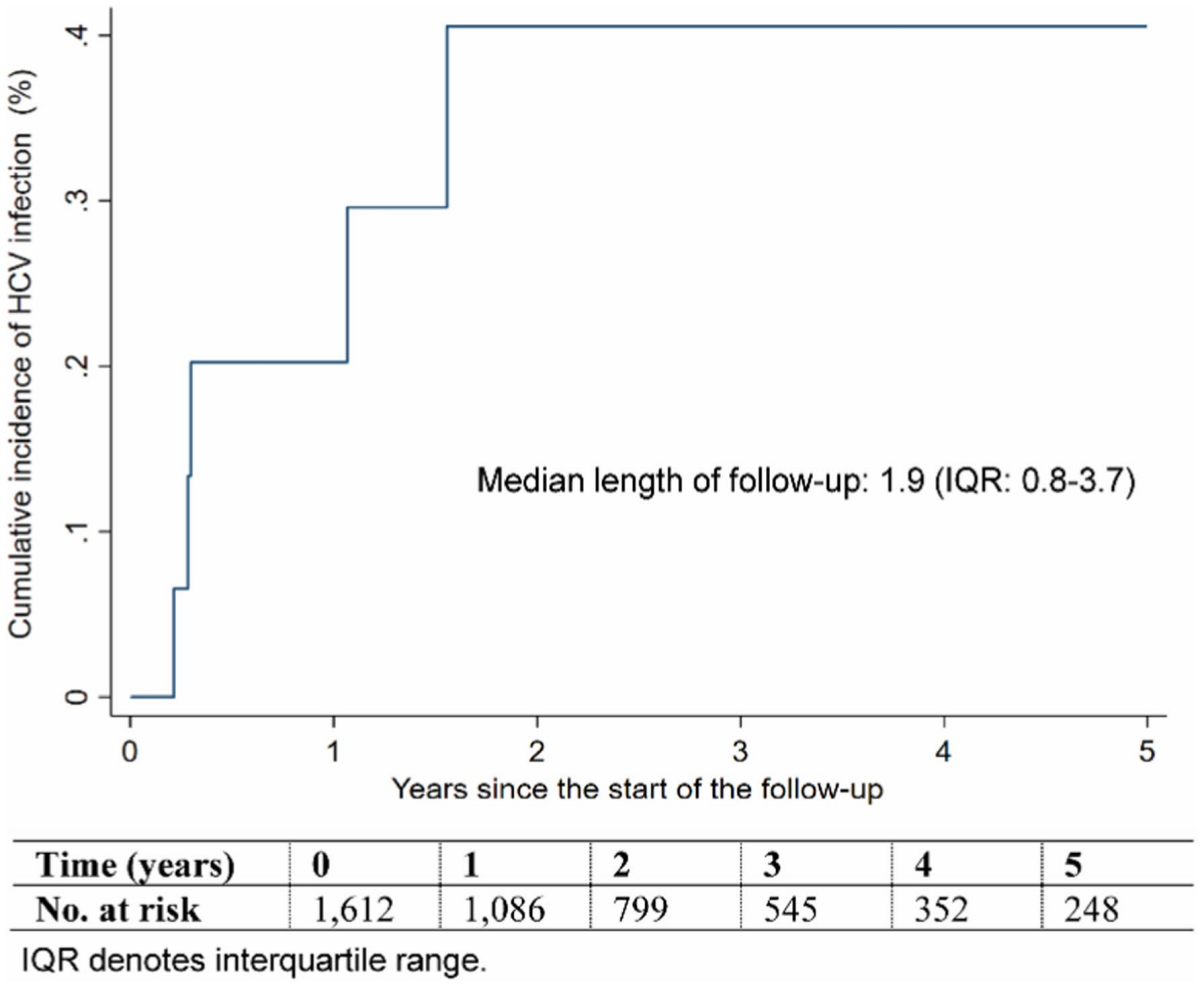
Cumulative incidence of HCV infection during follow-up.

Supplementary Table S4 provides the baseline characteristics of the HCV incidence study cohort. The median duration of follow-up for this cohort was 1.9 years (IQR: 0.8–3.7). During the follow-up period of 4,190.4 person-years, there were a total of 5 incident HCV infections, as shown in Figure 3 and Supplementary Table S5.

The cumulative incidence of HCV infection in this cohort, after 5 years of follow-up, was estimated at 0.41% (95% CI: 0.17–0.99%), as illustrated in Figure 3. The incidence rate of infection was calculated at 1.19 (95% CI: 0.50–2.87) per 1,000 person-years.

## Discussion

HCV Ab prevalence in the study population was found to be substantial at ~5%. This prevalence level is 10 times higher than the estimated HCV Ab prevalence in the general population, which was previously reported at only 0.3% based on a meta-analysis (13). The HCV incidence rate was also found to be substantial at 1 per 1,000 person-years, which is 100 times higher than the threshold for declaring infection elimination set at an incidence rate of 1 per 100,000 person-years (33, 34). Moreover, it is 20 times higher than the WHO target of achieving an incidence of ≤5 per 100,000 people per year by 2030 (11). However, the results suggest a potential decrease in HCV prevalence over time, which aligns with the overall trend observed in the MENA region (35, 36).

Although it is not surprising to observe a significant level of prevalence and incidence among a population that actively seeks HCV testing, these results are unexpected for Jordan, a country thought to have one of the lowest levels of HCV infection (13, 35, 36). These findings suggest the presence of core groups within the population who are actively acquiring and transmitting HCV infection. It is critical to describe and identify these core groups if Jordan aims to achieve the WHO targets for HCV infection by 2030. Additional efforts are necessary to address the underlying factors contributing to the higher prevalence and incidence, and to implement targeted interventions among these core groups in order to effectively control the spread of HCV in the country.

Due to the nature of the study, which relied on a testing database without associated behavioral or clinical data, it is not possible to directly identify the modes of HCV exposure. However, the observed associations provide insights into potential modes of exposure. The rapid increase in HCV infection likelihood with age, particularly among individuals aged 50 years and older, suggests a potential role for medical care in HCV exposures. A recent study from Jordan, conducted on a sample of 48 patients with chronic HCV infection, reported exposures including major surgeries, blood transfusions, *hijama* (a traditional medicine practice of cupping), invasive dental procedures, and hemodialysis (23). This is consistent with evidence from the MENA region, where HCV infection has been associated with healthcare-related exposures such as blood transfusions, hemodialysis, surgical procedures, dental work, and medical injections (12, 24, 27, 35, 37, 38).

Males had a higher likelihood of HCV infection compared to females, suggesting that injecting drug use may play a significant role as a mode of exposure in the study population. This practice is much more prevalent among men than women in the MENA region (25, 26, 39, 40). Despite the limited testing sample of individuals aged 0–9 years, a measurable HCV Ab prevalence of 1.9% was observed, raising the possibility of mother-to-child transmission as a mode of exposure (41). This mode of transmission is rare in Jordan due to the low HCV prevalence in the general population (13, 42, 43), but one case of suspected mother-to-child transmission was identified in the sample of 48 patients with chronic HCV infection indicated above (23).

HCV Ab prevalence was higher outside the Amman governorate. However, the number of tests conducted outside Amman was considerably lower than that conducted within Amman. Consequently, the samples of individuals tested outside Amman may have been biased towards individuals at a higher risk of infection. While the reason for the higher prevalence of HCV outside the Amman governorate remains unknown, it is uncertain whether this may relate to a higher incidence of HCV healthcare-related exposures, potentially indicating less advanced medical care in those areas.

The HCV viremic rate in this study was 54%, which is lower than the estimated rate of 68% in the MENA region based on studies conducted prior to the availability of DAA treatment (29). This difference suggests that some individuals who were chronically infected with HCV may have already received treatment and successfully cleared the infection. However, the observed viremic rate in this study is still relatively high, indicating an unmet need for HCV DAA treatment in Jordan. There is a significant portion of individuals who are viremic and would benefit from access to effective antiviral therapy to eliminate the infection.

The findings underscore the importance of expanding HCV testing and treatment efforts in Jordan through an HCV treatment as prevention (HCV-TasP) approach (34, 44, 45). The results also emphasize the urgent need to identify and engage core groups at high risk of HCV acquisition through targeted interventions. It is further recommended to conduct a national population-based HCV survey to assess infection levels, similar to initiatives undertaken in other countries such as Egypt (13–15), by incorporating it into existing surveys like the Jordan Demographic and Health Survey (46). This comprehensive approach will enhance understanding of the HCV burden and facilitate the implementation of appropriate interventions to control the spread of HCV in Jordan.

Identifying individuals with chronic HCV infection who are unaware of their condition poses a significant challenge both in the MENA region and on a global scale (37). Nevertheless, the findings offer a promising avenue to tackle this issue. The extensive volume of HCV tests conducted by Biolab and other diagnostic laboratories with broad national coverage presents a unique opportunity to detect individuals carrying the infection and seamlessly link them to appropriate treatment and care through a national program.

It is estimated that around 20,000 individuals are chronically infected with HCV in Jordan (34). Remarkably, our study reveals that Biolab has identified over 1,000 individuals who tested positive for HCV antibodies (Table 1). Considering that more than half of them are viremic, it is evident that HCV testing at Biolab alone has successfully identified over 5% of all HCV chronic carriers in Jordan. This striking outcome underscores the effectiveness of implementing such testing approaches within diagnostic laboratories as a mean to detect HCV chronic carriers.

Furthermore, these diagnostic laboratories perform millions of tests annually for various health indicators. This extensive reach enables the implementation of targeted facility-based testing strategies, providing HCV testing to individuals at higher risk of infection. This can be accomplished by utilizing risk scores (47, 48), as recently demonstrated in Egypt (49). Exploring such approaches to combat HCV infection is needed, especially considering the limited funding available for HCV programming. It is essential to recognize that poorly designed testing strategies and ineffective testing campaigns can have negative financial implications and may weaken the political will to expand test-and-treat programs.

This study is subject to several limitations. Firstly, given the retrospective nature of the work, the available characteristics for the tested population lacked behavioral or clinical data. As a result, the interpretation of observed patterns and trends is constrained, limiting their generalizability. It is important to note that the tested population includes a subset of individuals at higher risk of HCV infection and does not represent the broader population of Jordan.

Secondly, the tracking of repeat HCV tests relied on linking them to the same patient number. However, there is a possibility that some individuals may have been registered more than once under different patient numbers, potentially introducing errors that could impact the estimates. While this is a theoretical possibility, it does not seem likely as the registration system is designed to provide an alert in case of an existing match for the patient. Duplicate registration could happen only if the patient intentionally provided incorrect information, but this is unlikely for HCV testing as individuals typically test through third-party referrals or as part of follow-up visits.

Thirdly, although the laboratory methods utilized in this study were based on high-quality, validated, and widely used commercial platforms, these assays may not achieve perfect sensitivity and specificity. Consequently, false-negative or false-positive test results are a possibility. However, the study employed a detailed algorithm to confirm the test outcomes (Supplementary Figure S1). This involved utilizing a second quality assay for confirmatory testing, requesting a new sample for retesting if necessary, and ultimately confirming the results through a reference laboratory. These rigorous measures significantly reduce the likelihood of false results and enhance the reliability of the study findings.

Despite these limitations, the study analyzed a large testing sample, enabling various analyses and generating different epidemiological results and inferences. The study has addressed significant gaps in evidence regarding HCV infection in Jordan.

In conclusion, this study highlighted substantial prevalence and incidence of HCV infection among the study population, consisting of individuals attending a widely distributed high throughput diagnostic laboratory for HCV testing (22). These findings appear to challenge the perception of Jordan as a country with very low HCV infection rates and underscore the existence of core groups actively involved in HCV acquisition and transmission. Targeted interventions aimed at these core groups are critical to meet the WHO targets for HCV infection by 2030. The high viremic rate observed emphasizes the immediate need for expanded HCV treatment efforts, employing an HCV-TasP approach, and offering linkage to DAA treatment immediately upon diagnosis of infection. To enhance our understanding of the HCV burden and implement appropriate interventions, it is recommended to conduct a national population-based survey and utilize diagnostic laboratories for targeted testing strategies. Overcoming the challenges in identifying individuals with chronic HCV infection requires leveraging the wide reach of diagnostic laboratories and implementing innovative testing approaches. By doing so, we can effectively link infected individuals to necessary treatment and care, ultimately contributing to the control of HCV transmission in Jordan and the MENA region.

## Data availability statement

The dataset of this study is a property of Biolab Diagnostic Laboratories that was provided to the researchers for scientific research purposes through a restricted-access agreement that prevents sharing the dataset with a third party or publicly. The data are available upon reasonable request and under restricted access for preservation of confidentiality of patient data. Access can be obtained through a direct application for data access to Dr. Issa Abu-Dayyeh, Biolab Diagnostic Laboratories, E-mail: i.abudayyeh@biolab.jo. The raw data are protected and are not available due to data privacy laws. Data were available to authors through .csv files (no links/accession codes were available to authors). Aggregate data are available within the manuscript and its Supplementary material.

## Ethics statement

The studies involving humans were approved by Institutional Review Boards at Biolab and Weill Cornell Medicine–Qatar. The studies were conducted in accordance with the local legislation and institutional requirements. Written informed consent for participation was not required from the participants or the participants' legal guardians/next of kin in accordance with the national legislation and institutional requirements.

## Author contributions

IA-D: Conceptualization, Data curation, Methodology, Writing – original draft, Writing – review & editing. HC: Conceptualization, Formal analysis, Investigation, Methodology, Writing – original draft, Writing – review & editing. MG: Data curation, Writing – review & editing. TH: Data curation, Writing – review & editing. AA: Writing – review & editing. LA-R: Conceptualization, Funding acquisition, Investigation, Methodology, Project administration, Supervision, Writing – original draft, Writing – review & editing.
