# Supplementary material for: Patterns and trends of hepatitis C virus infection in Jordan: an observational study
Source: Front Public Health. 2023 Dec 7;11:1280427. doi: 10.3389/fpubh.2023.1280427 (PMC10749371; doi:10.3389/fpubh.2023.1280427)
Supplement: Supplementary file 1 [file Data_Sheet_1.docx]

Supplementary Material

**Table of Contents**

[**Figure S1. Illustration of the algorithm employed for HCV antibody testing.** 2](#_Toc143192574)

[**Table S1. STROBE checklist for cross-sectional studies.** 3](#_Toc143192575)

[**Table S2. STROBE checklist for cohort studies.** 5](#_Toc143192576)

[**Table S3. Characteristics of individuals with at least one HCV RNA test.** 7](#_Toc143192577)

[**Figure S2. Distribution of antibody titers (optical density values) among HCV antibody positive individuals.** 8](#_Toc143192578)

[**Figure S3. Distribution of HCV RNA viral load among HCV RNA positive individuals.** 9](#_Toc143192579)

[**Table S4. Baseline characteristics of HCV incidence cohort.** 10](#_Toc143192580)

[**Table S5. Incident HCV infections in the HCV incidence cohort.** 11](#_Toc143192581)

**Figure S1. Illustration of the algorithm employed for HCV antibody testing.**

**
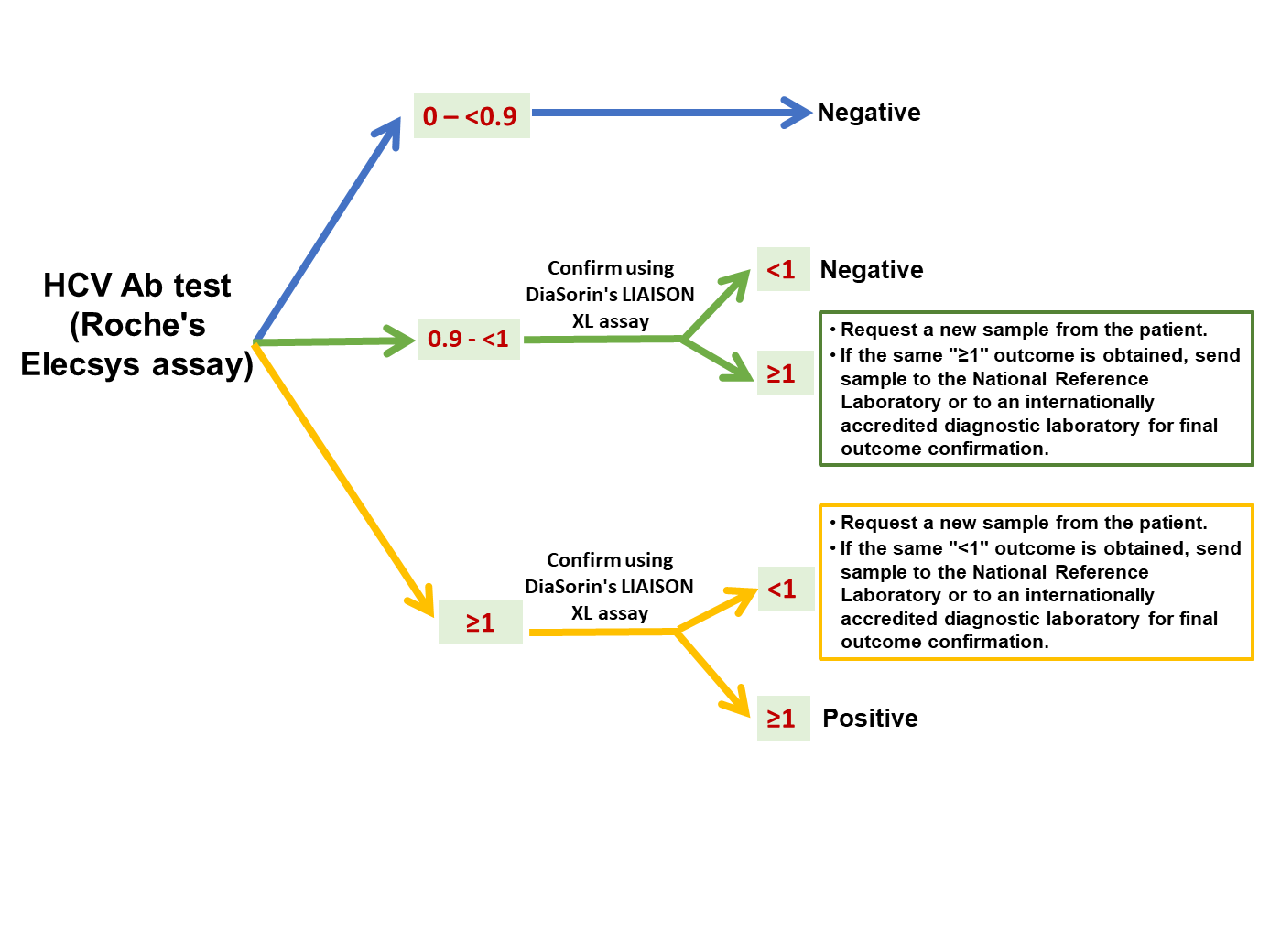
**

**Table S1. STROBE checklist for cross-sectional studies.**

|  | **Item No** | **Recommendation** | **Main Text** |
| --- | --- | --- | --- |
| **Title and abstract** | 1 | (*a*) Indicate the study’s design with a commonly used term in the title or the abstract | Abstract |
|  |  | (*b*) Provide in the abstract an informative and balanced summary of what was done and what was found | Abstract |
| **Introduction** | | |  |
| Background/rationale | 2 | Explain the scientific background and rationale for the investigation being reported | Introduction |
| Objectives | 3 | State specific objectives, including any prespecified hypotheses | Introduction |
| **Methods** | | |  |
| Study design | 4 | Present key elements of study design early in the paper | Methods (‘Study designs and analyses’) |
| Setting | 5 | Describe the setting, locations, and relevant dates, including periods of recruitment, exposure, follow-up, and data collection | Methods (‘Study population and data sources’ & ‘Study designs and analyses’) |
| Participants | 6 | (*a*) Give the eligibility criteria, and the sources and methods of selection of participants | Methods (‘Study population and data sources’ & ‘Study designs and analyses’) |
| Variables | 7 | Clearly define all outcomes, exposures, predictors, potential confounders, and effect modifiers. Give diagnostic criteria, if applicable | Methods (‘Study designs and analyses’, ‘Laboratory methods’, & ‘Statistical analysis’) |
| Data sources/ measurement | 8* | For each variable of interest, give sources of data and details of methods of assessment (measurement). Describe comparability of assessment methods if there is more than one group | Methods (‘Study population and data sources’, ‘Study designs and analyses’, ‘Laboratory methods’, & ‘Statistical analysis’) |
| Bias | 9 | Describe any efforts to address potential sources of bias | Methods (‘Study designs and analyses’, paragraph 2, & ‘Statistical analysis’, paragraph 2) |
| Study size | 10 | Explain how the study size was arrived at | Methods (‘Study designs and analyses’) & Figure 1 |
| Quantitative variables | 11 | Explain how quantitative variables were handled in the analyses. If applicable, describe which groupings were chosen and why | Methods (‘Laboratory methods’ & ‘Statistical analysis’), Tables 1-2, & Table S3 & Figures S1-S2 in Supplementary Material |
| Statistical methods | 12 | (*a*) Describe all statistical methods, including those used to control for confounding | Methods (‘Statistical analysis’) |
|  |  | (*b*) Describe any methods used to examine subgroups and interactions | Methods (‘Statistical analysis’) |
|  |  | (*c*) Explain how missing data were addressed | Methods (‘Study designs and analyses’, paragraph 1) |
|  |  | (*d*) If applicable, describe analytical methods taking account of sampling strategy | Methods (‘Study designs and analyses’, paragraph 2, & ‘Statistical analysis’, paragraph 2) |
|  |  | (*e*) Describe any sensitivity analyses | Not applicable |
| **Results** | | |  |
| Participants | 13* | (a) Report numbers of individuals at each stage of study—eg numbers potentially eligible, examined for eligibility, confirmed eligible, included in the study, completing follow-up, and analysed | Results (‘Study samples’) & Figure 1 |
|  |  | (b) Give reasons for non-participation at each stage |  |
|  |  | (c) Consider use of a flow diagram |  |
| Descriptive data | 14* | (a) Give characteristics of study participants (eg demographic, clinical, social) and information on exposures and potential confounders | Results (‘Study samples’), Table 1 & Table S3 in Supplementary Material |
|  |  | (b) Indicate number of participants with missing data for each variable of interest | Not applicable, see Methods (‘Study designs and analyses’, paragraph 1) |
| Outcome data | 15* | Report numbers of outcome events or summary measures | Results (‘HCV Ab prevalence’, ‘Associations with HCV Ab positivity’, & ‘HCV viremic rate’), Figure 2, Table 2, & Figures S1 & S2 in Supplementary Material |
| Main results | 16 | (*a*) Give unadjusted estimates and, if applicable, confounder-adjusted estimates and their precision (eg, 95% confidence interval). Make clear which confounders were adjusted for and why they were included | Results (‘HCV Ab prevalence’, ‘Associations with HCV Ab positivity’, & ‘HCV viremic rate’), Figure 2, Table 2, & Figures S1 & S2 in Supplementary Material |
|  |  | (*b*) Report category boundaries when continuous variables were categorized | Tables 1-2 & Table S3 in Supplementary Material |
|  |  | (*c*) If relevant, consider translating estimates of relative risk into absolute risk for a meaningful time period | Not applicable |
| Other analyses | 17 | Report other analyses done—eg analyses of subgroups and interactions, and sensitivity analyses | Not applicable |
| **Discussion** | | |  |
| Key results | 18 | Summarise key results with reference to study objectives | Discussion, paragraphs 1-10 |
| Limitations | 19 | Discuss limitations of the study, taking into account sources of potential bias or imprecision. Discuss both direction and magnitude of any potential bias | Discussion, paragraphs 11-13 |
| Interpretation | 20 | Give a cautious overall interpretation of results considering objectives, limitations, multiplicity of analyses, results from similar studies, and other relevant evidence | Discussion, paragraph 15 |
| Generalisability | 21 | Discuss the generalisability (external validity) of the study results | Discussion, paragraph 11 |
| **Other information** | | |  |
| Funding | 22 | Give the source of funding and the role of the funders for the present study and, if applicable, for the original study on which the present article is based | Funding |

**Table S2. STROBE checklist for cohort studies.**

|  | **Item No** | **Recommendation** | **Main Text** |
| --- | --- | --- | --- |
| **Title and abstract** | 1 | (*a*) Indicate the study’s design with a commonly used term in the title or the abstract | Abstract |
|  |  | (*b*) Provide in the abstract an informative and balanced summary of what was done and what was found | Abstract |
| **Introduction** | | | |
| Background/rationale | 2 | Explain the scientific background and rationale for the investigation being reported | Introduction |
| Objectives | 3 | State specific objectives, including any prespecified hypotheses | Introduction |
| **Methods** | | | |
| Study design | 4 | Present key elements of study design early in the paper | Methods (‘Study designs and analyses’) |
| Setting | 5 | Describe the setting, locations, and relevant dates, including periods of recruitment, exposure, follow-up, and data collection | Methods (‘Study population and data sources’ & ‘Study designs and analyses’) |
| Participants | 6 | (*a*) Give the eligibility criteria, and the sources and methods of selection of participants. Describe methods of follow-up | Methods (‘Study population and data sources’ & ‘Study designs and analyses’, paragraphs 1 & 4) |
|  |  | (*b*) For matched studies, give matching criteria and number of exposed and unexposed |  |
| Variables | 7 | Clearly define all outcomes, exposures, predictors, potential confounders, and effect modifiers. Give diagnostic criteria, if applicable | Methods (‘Study designs and analyses’, ‘Laboratory methods’, & ‘Statistical analysis’) |
| Data sources/ measurement | 8* | For each variable of interest, give sources of data and details of methods of assessment (measurement). Describe comparability of assessment methods if there is more than one group | Methods (‘Study population and data sources’, ‘Study designs and analyses’, ‘Laboratory methods’, & ‘Statistical analysis’) |
| Bias | 9 | Describe any efforts to address potential sources of bias | Methods (‘Study designs and analyses’, paragraph 4) |
| Study size | 10 | Explain how the study size was arrived at | Methods (‘Study designs and analyses’, paragraphs 1 & 4) & Figure 1 |
| Quantitative variables | 11 | Explain how quantitative variables were handled in the analyses. If applicable, describe which groupings were chosen and why | Methods (‘Laboratory methods’), Tables 1-2, & Table S4 in Supplementary Material |
| Statistical methods | 12 | (*a*) Describe all statistical methods, including those used to control for confounding | Methods (‘Statistical analysis’) |
|  |  | (*b*) Describe any methods used to examine subgroups and interactions | Methods (‘Statistical analysis’) |
|  |  | (*c*) Explain how missing data were addressed | Methods (‘Study designs and analyses’, paragraph 1) |
|  |  | (*d*) If applicable, explain how loss to follow-up was addressed | Methods (‘Study designs and analyses’, paragraph 4) |
|  |  | (*e*) Describe any sensitivity analyses | Not applicable |
| **Results** | | |  |
| Participants | 13* | (a) Report numbers of individuals at each stage of study—eg numbers potentially eligible, examined for eligibility, confirmed eligible, included in the study, completing follow-up, and analysed | Results (‘HCV incidence rate’) & Figure 1 |
|  |  | (b) Give reasons for non-participation at each stage |  |
|  |  | (c) Consider use of a flow diagram |  |
| Descriptive data | 14 | (a) Give characteristics of study participants (eg demographic, clinical, social) and information on exposures and potential confounders | Table S4 in Supplementary Material |
|  |  | (b) Indicate number of participants with missing data for each variable of interest | Not applicable, see Methods (‘Study designs and analyses’, paragraph 1) |
|  |  | (c) Summarise follow-up time (eg, average and total amount) | Results (‘HCV incidence rate’) & Figure 3 |
| Outcome data | 15 | Report numbers of outcome events or summary measures over time | Results (‘HCV incidence rate’), Figure 3, & Table S5 in Supplementary Material |
| Main results | 16 | (a) Give unadjusted estimates and, if applicable, confounder-adjusted estimates and their precision (eg, 95% confidence interval). Make clear which confounders were adjusted for and why they were included | Results (‘HCV incidence rate’), Figure 3, & Table S5 in Supplementary Material |
|  |  | (b) Report category boundaries when continuous variables were categorized | Table S4 in Supplementary Material |
|  |  | (c) If relevant, consider translating estimates of relative risk into absolute risk for a meaningful time period | Not applicable |
| Other analyses | 17 | Report other analyses done—eg analyses of subgroups and interactions, and sensitivity analyses | Not applicable |
| **Discussion** | | | |
| Key results | 18 | Summarise key results with reference to study objectives | Discussion, paragraphs 1-10 |
| Limitations | 19 | Discuss limitations of the study, taking into account sources of potential bias or imprecision. Discuss both direction and magnitude of any potential bias | Discussion, paragraphs 11-13 |
| Interpretation | 20 | Give a cautious overall interpretation of results considering objectives, limitations, multiplicity of analyses, results from similar studies, and other relevant evidence | Discussion, paragraph 15 |
| Generalisability | 21 | Discuss the generalisability (external validity) of the study results | Discussion, paragraph 11 |
| **Other information** | | | |
| Funding | 22 | Give the source of funding and the role of the funders for the present study and, if applicable, for the original study on which the present article is based | Funding |

**Table S3. Characteristics of individuals with at least one HCV RNA test.**

| **Characteristics** | **N (%)**  **N=1,450** |
| --- | --- |
| Median age (IQR)—years | 42.2 (30.8-52.5) |
| Age (years) |  |
| 0-9 | 33 (2.3) |
| 10-19 | 32 (2.2) |
| 20-29 | 233 (16.1) |
| 30-39 | 347 (23.9) |
| 40-49 | 342 (23.6) |
| 50-59 | 275 (19.0) |
| 60-69 | 122 (8.4) |
| 70+ | 66 (4.6) |
| Sex |  |
| Female | 475 (32.8) |
| Male | 975 (67.2) |
| Governorate |  |
| Amman | 1,315 (90.7) |
| Aqaba | 31 (2.1) |
| Balqaa | 4 (0.3) |
| Irbid | 27 (1.9) |
| Karak | 14 (1.0) |
| Maan | 6 (0.4) |
| Madaba | 0 (0.0) |
| Tafila | 1 (0.1) |
| Zarqa | 52 (3.6) |
| Year of first HCV RNA test |  |
| 2010 | 113 (7.8) |
| 2011 | 218 (15.0) |
| 2012 | 153 (10.6) |
| 2013 | 76 (5.2) |
| 2014 | 100 (6.9) |
| 2015 | 91 (6.3) |
| 2016 | 107 (7.4) |
| 2017 | 101 (7.0) |
| 2018 | 84 (5.8) |
| 2019 | 143 (9.9) |
| 2020 | 83 (5.7) |
| 2021 | 91 (6.3) |
| 2022 | 65 (4.5) |
| 2023 | 25 (1.7) |
| Proportion with |  |
| Only 1 test | 1,349 (93.0) |
| 2 tests | 53 (3.7) |
| 3 tests | 24 (1.7) |
| 4 tests | 11 (0.8) |
| 5+ tests | 13 (0.9) |
| HCV RNA |  |
| At least one positive RNA test | 645 (44.5) |
| All negative tests | 805 (55.5) |

HCV denotes hepatitis C virus and IQR, interquartile range.

**Figure S2. Distribution of antibody titers (optical density values) among HCV antibody positive individuals.**

**Figure S3. Distribution of HCV RNA viral load among HCV RNA positive individuals.**

**Table S4. Baseline characteristics of HCV incidence cohort.**

| **Characteristics** | **N (%)**  **N=1,612** |
| --- | --- |
| Median age (IQR)—years | 31.3 (26.1-38.8) |
| Age (years) |  |
| 0-9 | 18 (1.1) |
| 10-19 | 46 (2.8) |
| 20-29 | 655 (40.6) |
| 30-39 | 525 (32.6) |
| 40-49 | 192 (11.9) |
| 50-59 | 110 (6.8) |
| 60-69 | 35 (2.2) |
| 70+ | 31 (1.9) |
| Sex |  |
| Female | 796 (49.4) |
| Male | 816 (50.6) |
| Governorate |  |
| Amman | 1,582 (98.1) |
| Aqaba | 2 (0.1) |
| Balqaa | 12 (0.7) |
| Irbid | 14 (0.9) |
| Karak | 0 (0.0) |
| Maan | 0 (0.0) |
| Madaba | 0 (0.0) |
| Tafila | 0 (0.0) |
| Zarqa | 2 (0.1) |
| Year of first HCV RNA test |  |
| 2010 | 95 (5.9) |
| 2011 | 107 (6.6) |
| 2012 | 98 (6.1) |
| 2013 | 142 (8.8) |
| 2014 | 158 (9.8) |
| 2015 | 167 (10.4) |
| 2016 | 182 (11.3) |
| 2017 | 136 (8.4) |
| 2018 | 129 (8.0) |
| 2019 | 143 (8.9) |
| 2020 | 99 (6.1) |
| 2021 | 77 (4.8) |
| 2022 | 69 (4.3) |
| 2023 | 10 (0.6) |
| Proportion with |  |
| 2 tests | 1,279 (79.3) |
| 3 tests | 218 (13.5) |
| 4 tests | 60 (3.7) |
| 5+ tests | 55 (3.4) |

HCV denotes hepatitis C virus and IQR, interquartile range.

**Table S5. Incident HCV infections in the HCV incidence cohort.**

| **Patient** | **First (negative) test at baseline** | | **Positive test associated with incidence of infection** | |
| --- | --- | --- | --- | --- |
|  | **Test date** | **HCV antibody titer** | **Test date** | **HCV antibody titer** |
| 1 | 7-Dec-13 | 0.773 | 31-May-14 | 1.58 |
| 2 | 22-Mar-14 | 0.049 | 25-Aug-14 | 30.55 |
| 3 | 31-Jul-16 | 0.85 | 11-Sep-19 | 1.33 |
| 4 | 5-Sep-16 | 0.046 | 24-Oct-18 | 143.5 |
| 5 | 1-Jul-20 | 0.035 | 24-Jan-21 | 68.5 |
